# Potential regulatory network in the PSG10P/miR‐19a‐3p/IL1RAP pathway is possibly involved in preeclampsia pathogenesis

**DOI:** 10.1111/jcmm.13985

**Published:** 2018-10-28

**Authors:** Nan Wang, Ruizhen Li, Min Xue

**Affiliations:** ^1^ Department of Obstetrics and Gynecology The Third Xiangya Hospital of Central South University Changsha China

**Keywords:** IL1RAP, miR‐19a‐3p, preeclampsia pathogenesis, PSG10P

## Abstract

Preeclampsia (PE), a pregnancy‐specific disorder, is a leading cause of perinatal maternal‐fetal mortality and morbidity. Impaired cell migration and invasion of trophoblastic cells and an imbalanced systemic maternal inflammatory response have been proposed as potential mechanisms of PE pathogenesis. Comparative analysis between PE placentas and normal placentas profiled differentially expressed miRNAs, lncRNAs, and mRNAs, including miR‐19a‐3p (miRNA), *PSG10P* (lncRNA), and *IL1RAP* (mRNA). This study was conducted to investigate their potential roles in PE pathogenesis. The expression of miR‐19a‐3p, PSG10P, and IL1RAP was examined in PE and normal placentas using RT‐qPCR. An in vitro experiment was performed in human trophoblast HET8/SVneo and TEV‐1 cells cultured in normoxic and hypoxic conditions. MiR‐19a‐3p targets were identified using Targetscan, miRanda, and PicTar analysis as well as luciferase reporter assays. The mouse model of PE was conducted using sFlt‐1 for in vivo tests. Lower levels of miR‐19a‐3p, but higher levels of PSG10P and IL1RAP were observed in PE placentas and the trophoblast cells in hypoxia. Luciferase reporter assays confirmed that PSG10P and IL1RAP were both direct targets of miR‐19a‐3p. Exposure to hypoxia inhibited cell viability, migration, and invasion of HET8/SVneo and TEV‐1 cells. Knocking out PSG10P and IL1RAP or overexpressing miR‐19a‐3p rescued the inhibition caused by hypoxia. In vivo experiments showed that IL1RAP promoted the expression of caspase‐3, a key apoptosis enzyme, but inhibited MMP9, which is responsible for degrading the extracellular matrix, suggesting a significant role of IL1RAP in cell proliferation, migration, and invasion. miR‐19a‐3p, PSG10P, and IL1RAP were all found to be involved in PE pathogenesis. With a common targeting region in their sequences, a regulatory network in the PSG10P/miR‐19a‐3p/IL1RAP pathway may contribute to PE pathogenesis during pregnancy.

## 1 INTRODUCTION

Preeclampsia (PE) is a pregnancy‐specific disease characterized by hypertension (defined as systolic blood pressure P140 mm Hg or diastolic blood pressure P90 mm Hg) and proteinuria (>300 mg protein in a 24‐hour urine specimen and/or protein to creatinine ratio of >0.30) that occurs after 20 weeks of pregnancy.^1^, ^2^ PE affects 3%‐8% of pregnancies worldwide and triggers problems in the liver, kidney, brain, and the clotting system during pregnancy.^3^ As a result, PE is still one of the leading causes of maternal and fetal morbidity and mortality.^4^ Growing evidence supports that PE results from abnormal placental development and function,^3^, ^4^ however, the pathophysiological mechanisms of PE remain poorly understood. In normal pregnancies, cytotrophoblasts (CTBs) migrate through the decidua and myometrium in order to invade the maternal spiral arteries of the endothelium and tunica media.^5^ Inadequate invasion possibly results in reduced uteroplacental perfusion pressure (RUPP) and placental ischaemia/hypoxia.^6^ Ischaemia‐induced oxidative stress also leads to the apoptosis and necrosis of the placenta as well as the release of several placental factors, resulting in endothelial dysfunction, and systemic inflammation.^7^ In normal pregnancies, placentas promote maternal tolerance to fetal antigens partly through producing immunosuppressive molecules, inhibiting chemokine expression and complement activation, entrapping professional antigen‐presenting cells, and excluding immune cells.^8^, ^9^, ^10^ However, in patients with PE, abnormal placentation is associated with an exaggerated systemic maternal inflammatory response, which contributes to adverse pregnancy outcomes.^8^, ^9^, ^10^

Upon deciphering the human genome, large‐scale investigations of non‐coding RNAs (ncRNAs), including miRNAs and lncRNAs, have been conducted in many fields. MicroRNAs (miRNAs) are small (18‐24 nt) endogenous non‐coding single‐stranded RNAs. MiRNAs regulate mRNA, which encode proteins that modulate cellular functions, by binding to the untranslated region (3′UTR) of target mRNAs. As a result, target gene expression can be regulated post‐transcriptionally through degradation of target mRNAs.^11^ Therefore, miRNAs play important roles in physiological homoeostasis in health and pathophysiological dysregulation in disease.^11^, ^12^ To date, more than 1000 miRNAs have been validated in humans, among which more than 600 miRNAs are expressed in the human placenta.^13^ Abnormal expression of specific miRNAs has been reported in various studies of human diseases, including PE.^11^ Comparative analysis between PE placentas and normal placentas identified 11 up‐regulated and 23 down‐regulated miRNAs.^14^ Among them, Has‐miR‐19a was down‐regulated by 0.488‐fold. Transcriptional downregulation of miR‐19a was also observed under oxidative stress.^15^ Overexpression of miR‐19a protects endothelial cells from lipopolysaccharide‐induced apoptosis^16^ and enhanced cell invasion and metastasis.^17^ These findings imply a putative implication of miR‐19a in the physiological pathological mechanisms of PE.

LncRNAs (long non‐coding RNAs) are non‐coding RNAs that are longer than 200 nt in length. LncRNAs can regulate gene expression potentially through complementary binding or as co‐regulators.^18^ Dysregulation of lncRNAs has been associated with many human diseases.^18^ To better understand the expression of lncRNAs and their biological functions in PE, an expression profile of lncRNAs of PE placenta was sequenced by He et al.^19^ In the GSE50783 dataset, lncRNA‐PSG10P was significantly up‐regulated in PE placentas compared to normal placentas.^19^ Pregnancy‐specific glycoproteins (PSGs) are the most abundant trophoblastic proteins in maternal blood during pregnancy.^20^ *PSG10P* is a non‐coding pseudogene.

Additionally, significant overexpression of IL‐1 receptor accessory protein (*IL1RAP*) mRNA was also detected in the GSE50783 dataset. Interleukin‐1 (IL‐1) family cytokines are potent components of the innate immune system.^19^ Some IL‐1 family members bind the IL‐1 receptor 1 (IL‐1R1), which then complexes with the IL‐1RAP, triggering inflammatory signaling.^21^, ^22^ The interplay between IL1RAP and IL‐1R1 is indispensable for transmission nb IL‐1 signaling. Degradation of IL1RAP induced by ubiquitination interferes with the NF‐κB activation induced by IL‐1β and the inflammatory and immune responses.^23^ Therefore, the critical role of IL1RAP in IL‐1 signal transduction suggests the involvement of IL1RAP in various biological and pathological functions affected by IL‐1 that represents an important pro‐inflammatory cytokine.

Bioinformatic analysis revealed that PSG10P can promote the expression of IL1RAP by sponging miR‐19a‐3p. Many studies have been performed on the interactions among RNA molecules, such as interactions between lncRNA‐miRNA and miRNA‐mRNA.^24^, ^25^ Here, we focused on the interaction between PSG10P‐miR‐19a‐3p and miR‐19a‐3p‐*IL1RAP*. In addition, we hypothesized that the PSG10P/miR‐19a‐3p/IL1RAP network regulates the growth and invasion of trophoblastic cells under hypoxia. This hypothesis was tested in two trophoblast cell lines, namely HTR‐8/SVneo and TEV‐1. The potential effect of miR‐19a‐3p on PE occurrence was further investigated in a mouse model of PE to better understand the potential pathogenesis of PE.

## 2 MATERIAL AND METHODS

### 2.1 Sample collection

From March 2017 to August 2018, placentas were collected through cesarean delivery at the maternity unit in the third Xiangya Hospital of Central South University (Changsha, China). Placental tissue and blood samples from patients with PE (n = 20) and normal controls (n = 20) were collected during delivery. The tissues and the serum separated from the blood were immediately frozen using liquid nitrogen and then stored at −80°C. All protocols used in this study were approved by the Research Medical Ethics Committee of the third Xiangya Hospital of Central South University. All women were informed of the research nature of our study and provided informed consent. The clinical data from all patients and normal controls are shown in Table 1.

**Table 1:** Clinical information of PE patients and health controls

Patient number	Age (years)	Gestational weeks	Urine protein (mg/24 h)	MAP(mm Hg)
N1	26	38	–	95.25
N2	28	39 + 3	–	85.43
N3	31	39 + 2	–	89.36
N4	29	40 + 3	–	92.1
N5	34	39	–	96.17
N6	22	37 + 1	–	85.83
N7	30	35 + 6	–	89.31
N8	32	37	–	86.62
N9	27	38 + 4	–	97.86
N10	25	37 + 5	–	92.87
N11	23	39 + 5	–	88
N12	32	39 + 3	–	106
N13	24	40 + 5	–	84
N14	29	40 + 3	–	97
N15	27	38 + 4	–	103
N16	26	39	–	107
N17	30	38 + 3	–	99
N18	27	39 + 4	–	75
N19	25	40 + 1	–	105
N20	23	40 + 4	–	76
P1	33	38 + 1	4090	136.37
P2	25	37 + 2	1135	122.63
P3	34	39	3931	113.05
P4	27	38 + 2	1240	127.17
P5	24	37 + 6	2037	119.33
P6	28	37	162	121.67
P7	33	35 + 2	1536	131.4
P8	23	39 + 4	2493	115.33
P9	31	38 + 1	2499	118.2
P10	28	40 + 5	2803	117.33
P11	24	38 + 1	2018	120.67
P12	25	41 + 3	1764	112.67
P13	27	36 + 3	3421	114.57
P14	29	37 + 2	2763	119.16
P15	35	38	2437	134.58
P16	32	38 + 6	3058	111.85
P17	31	39 + 2	3991	120.68
P18	27	37 + 3	2643	136.41
P19	29	38 + 4	1726	138.54
P20	35	37 + 4	3576	126.08

N: normal controls; P: pre‐eclampsia patients.

### 2.2 RNA extraction and quantitative PCR (RT‐qPCR)

Total RNA, including small RNA, was isolated from tissue samples or cells using a mirVana miRNA kit (Thermo Fisher Scientific, Waltham, MA, USA) according to the manufacturer's instructions. The cDNA was synthesized using the High Capacity RNA‐to‐cDNA Master Mix (Life Technologies, Carlsbad, CA, USA). The primers used in the qPCR experiments are shown in Table 2. The reaction was performed on an ABI 7300 Real‐Time PCR System (Applied Biosystems, Foster City, CA, USA) using the SYBR ExScript RT‐PCR kit (TaKaRa, Dalian, China) according to the manufacturer's instructions. The qRCR thermal cycling was performed with a denaturation step of 30 seconds at 95°C, followed by 40 cycles at 95°C (5 seconds) and 60°C (30 seconds). The expression levels of miRNAs and genes were normalized to those of U6 and *GAPDH*, respectively, using the comparative threshold cycle (2^−ΔΔCT^) method.

**Table 2:** The primer sequences of the related genes

Gene	Sequence of primers (5′‐3′)
hsa‐PSG10P	F:5′‐AGGAAGGACAGCACAGCCTA‐3′
	R:5′‐TGGTAGGTGGGTTCCAGAAG‐3′
hsa‐miR‐19a‐3p	F:5′‐ACACTCCAGCTGGGTGTGCAAATCTATGCAA‐3′
	R:5′‐CTCAACTGGTGTCGTGGAGTCGGCAATTCAGTTGAGTCAGTTTT‐3′
mmu‐miR‐19a‐sp	F:5′‐ACACTCCAGCTGGGTGTGCAAATCTATGCAA‐3′
	R:5′‐CTCAACTGGTGTCGTGGAGTCGGCAATTCAGTTGAGTCAGTTTT‐3′
hsa‐IL1RAP	F:5′‐GGGGACTAGACACCATGAGG‐3′
	R:5′‐ACCAGATCAGAGTAAGGCCAG‐3′
hsa‐INHBA	F:5′‐CCTCCCAAAGGATGTACCCAA‐3′
	R:5′‐CTCTATCTCCACATACCCGTTCT‐3′
hsa‐TREM1	F:5′‐CTTGGCAGATAATAAGGGACGG‐3′
	R:5′‐CGGACGCGCAGTAAACCAT‐3′
hsa‐PSG11	F:5′‐CCTACCACTGCCCAAGTCAT‐3′
	R:5′‐ATTCTGGATCAGCAGGGATG‐3′
hsa‐RASEF	F:5′‐TTCCCCTCAACCTCTAGGCTA
	R:5′‐CAACTTCACAATTTGTCCTCTGC
U6	F:5′‐CTCGCTTCGGCAGCACA‐3′
	R:5′‐AACGCTTCACGAATTTGCGT‐3′
GAPDH	F:5′‐GGAGCGAGATCCCTCCAAAAT‐3′
	R:5′‐GGCTGTTGTCATACTTCTCATGG‐3′

### 2.3 Western blot analysis

Total proteins from frozen placentas were extracted by homogenizing and lysing in RIPA lysis buffer (Beyotime, Shanghai, China) supplemented with phenylmethanesulfonyl fluoride (PMSF, Beyotime). Total protein from HTR‐8/SVneo and TEV‐1 cells were lysed in 200 μl SDS lysis buffer (Beyotime) after seeding in 24‐well plates, then denatured at 100°C for 10 minutes. After total protein concentrations were determined using the BCA Reagent Kit (Beyotime), 20 μg protein from each sample was separated by a 10% or 12% sodium dodecyl sulfate–polyacrylamide electrophoresis (SDS‐PAGE) and transferred to a polyvinylidene fluoride (PVDF) blotting membrane (Bio‐Rad, Hercules, CA, USA). The membranes were first incubated in blocking buffer with non‐fat dry milk (Yili Milk Company, Inner Mongolia, China) at room temperature for 2 hours, and then hybridized with an anti‐IL1RAP antibody (1:1000 dilution; ab8110, Abcam, Cambridge, UK), anti‐Caspase‐3 antibody (1:1000 dilution; 19677‐1, Proteintech, Rosemont, IL, USA), anti‐matrix metalloproteinase (MMP)‐9 antibody (1:400 dilution, SC‐21733, Santa Cruz Biotechnology, Santa Cruz, CA, USA), or anti‐GAPDH antibody (Dilution 1:2000, SC‐365062, Santa Cruz Biotechnology) at 4°C overnight. Secondary biotin‐conjugated antibodies were added to visualize the primary antibodies followed by an avidin/biotin/peroxidase complex (Vectastain ABC Elite kit; Vector Laboratories Inc, Burlingame, CA, USA) and substrate (Vector NovaRED, Vectastain). Densitometric analysis from Quantity One software (Bio‐Rad) was used to quantify the band intensities representing protein expression levels.

### 2.4 Cell culture and treatments

HTR‐8/SVneo and TEV‐1 cells were obtained from ATCC (American Type Culture Collection, Manassas, VA, USA) and cultured in RPMI‐1640 medium (Life Technologies) containing 10% fetal bovine serum (Invitrogen, Carlsbad, CA, USA), and antibiotics (100 U/mL penicillin and 100 μg/mL streptomycin). In normal conditions, cells were cultured in 75‐cm^2^ plastic flasks at 37°C in a mixture of 5% CO_2_, 20% O_2_, and 75% N_2_ air atmosphere. While in hypoxic treatment, cells were cultured in an AW200SG hypoxic workstation (ELECTROTEK, Shipley, UK) with a continuous flow of a humidified mixture of 2% O_2_, 5% CO_2_, and 93% N_2_ at 37°C. Media were changed every 2‐3 days for both conditions. Cell viabilities were examined at 0, 12, 24, and 48 hours using a cell counting kit‐8 (CCK‐8, Dojindo Laboratories, Mashiki‐machi, Japan). The relative cell viability was calculated by comparing cell number at later time points (12, 24, and 48 hours) to that at the starting point (0 hour).

### 2.5 Cell transfection

HTR‐8/SVneo and TEV‐1 cells (1 × 10^5^ cells/well) were seeded in 6‐well plates. Small hairpin RNAs (shRNAs) targeting PSG10P (shRNA‐PSG10P) and IL1RAP (shRNA‐IL1RAP), the inhibitor and mimic oligonucleotides for hsa‐miR‐19a‐3p, and their negative controls were constructed by GenePharma company (Shanghai, China). ShRNA‐PSG10P, shRNA‐IL1RAP, and relevant controls were transfected into HTR‐8/SVneo and TEV‐1 cells using Lipofectamine 2000 (Invitrogen Life Technologies Company, Carlsbad, CA, USA) according to the manufacturer's instructions. The inhibitor and mimic oligonucleotides for hsa‐miR‐19a‐3p and their negative controls were transfected into cells using Lipofectamine RNAi Max (Thermo Fisher Scientific, Waltham, MA, USA). Cells were harvested at 48 hours for future analysis.

### 2.6 Luciferase reporter assay

To identify the putative binding site (seed sequence) of miR‐19a at the PSG10P and IL1RAP 3′UTR, bioinformatics analysis was performed using Targetscan, miRanda, and PicTar. The wild‐type (WT) and mutant (MT) target sites at the PSG10P and IL1RAP 3′UTR were amplified by PCR and then cloned into a pmirGLO vector (XhoI and NotI restriction enzyme sites; Promega, Madison, WI, USA). The 293 cells (1 × 10^4^ cells/well) were seeded in 12‐well plates and transfected with the WT or MT PSG10P and IL1RAP 3′UTR constructs together with either has‐miR‐19a‐3p mimics or negative control using Lipofectamine 2000 (Invitrogen). Cells were harvested at 48 hours and the activity of firefly luciferase was normalized to that of Renilla luciferase.

### 2.7 Transwell cell invasion assay

A Transwell assay was performed to examine the invasion ability of cells. HTR‐8/SVneo and TEV‐1 cells (2 × 10^5^ cells) were plated into the upper Transwell chamber (8 μm pore size; Corning; Corning, NY, USA). The Transwell plate was incubated at 37°C in a humidified 5% CO_2_ atmosphere for 48 hours. Non‐invading cells remaining on the surface of the top upper membrane were removed with a cotton‐tipped swab. Invading cells on the surface of the reverse membrane were blocked with ice‐cold methanol for 30 minutes and stained with 0.1% crystal violet. The stained cells were visualized by confocal microscopy and counted in 10 random fields per chamber (200× magnification).

### 2.8 Scratch assay

HTR‐8/SVneo and TEV‐1 cells were seeded in 6‐well plates from both hypoxic treatment and normoxic control. A scratch line was made on the cell monolayer using a 1‐mL pipette tip. FBS‐free culture medium was used in the subsequent cell culture. At 48 hours after scratching, microscopic images of the same area were captured. Cell migration was calculated based on the distance between the initial and final positions.

### 2.9 RNA pull‐down assay

An RNA pull‐down assay was performed to selectively extract the RNA(PSG10P)‐RNA (miR‐19a‐3p) complex at 48 hours from transfected samples. Biotinylated RNA probes (Bio‐miR‐NC, Bio‐miR‐19a‐3p and Bio‐miR‐19a‐3p‐Mut) were incubated with the cell lysates and purified using streptavidin‐coupled magnetic beads according to the instructions of the Pierce Magnetic RNA Pull‐Down Kit (Rockford, IL, USA). RNA‐RNA complexes were then eluted using the salt solution and purified using TRIzol (Pierce). The enrichment of PSG10P was quantified using qPCR as described above.

### 2.10 Animal treatment

The animal experiment was approved by the Ethical Committee for Animal Research of Central South University and adhered to National Institutes of Health Guidelines for the care and use of animals. Protein sFlt‐1 (Recombinant mouse VEGF R1/Flt‐1 Fc Chimera, Cat. No.7756‐FL‐050, Bio‐Rad) was used to establish PE pregnant mice. From day 7 of gestation, 3.7 μg/kg/day sFlt‐1 (in sterile saline) was injected into mice tail veins for 6 days. Among 36 PE mice, 12 mice were randomly selected for mmu‐miR‐agomir treatment as negative controls (PE+NC), 12 for mmu‐miR‐19a‐3p agomir treatment (PE+Agomir), and 12 as PE controls (PE). The mmu‐miR‐19a‐3p agomir (GenePharma) was used to increase miR‐19a‐3p expression in PE mice. Both mmu‐miR‐agomir and mmu‐miR‐19a‐3p agomir were injected into mice tail veins on day 13 of gestation at a rate of 100 μL/day for 6 days. In addition, another 12 normal pregnant mice were treated with saline solution as controls (Nor).

### 2.11 Data and tissues collection

Non‐invasive tail‐cuff blood pressure (BP) was measured in conscious mice using a non‐invasive automated sphygmomanometer (BP‐98A, Softron, Beijing, China). Mice were incubated at 38°C for 15 minutes to stabilize their physical signs and to expand their local vessels sufficiently. The pressurized sensor was placed in their tail, then blood pressure was measured after mice had calmed down for a few seconds. The average of five continuous measurements was taken as the final record for each mouse. To ensure relatively stable results, all BP measurements were performed at the same time every day during the experimental period. The protein concentrations were measured on day 19 of gestation. Mice were euthanized under anesthesia by chloral hydrate overdose. The urine was collected for further analysis of the protein concentration by the Bradford method using a Protein Quantification Kit (Beyotime). Placentas were collected for RT‐qPCR and western blot assays as described above and/or the following immunohistochemical analysis.

### 2.12 Immunohistochemical staining

The placenta tissues were embedded in paraffin and sectioned into 4‐μm slides. Then, slides were deparaffinized and hydrated. After inactivating endogenous peroxidase in 3% hydrogen peroxide, slides were retrieved in citric acid buffer (pH 6.0) by microwaving for 15 minutes. Slides were blocked in normal goat serum (Invitrogen) for 30 minutes at room temperature and incubated with an anti‐Ki67 antibody (Abcam) overnight at 4°C. Samples were then washed with TBST (Invitrogen) and incubated with the appropriate secondary antibody (Abcam) for 2 hours at 37°C. Sections were then washed with TBST and stained using a DAB Detection Kit (Solarbio, Beijing, China). Finally, sections were counterstained with hematoxylin. Section images were captured and analysed using Motic Images Advanced 3.2 software (Motic, XiaMen, China).

### 2.13 Statistical analysis

Data were analysed using SPSS 12.0 software (SPSS, Inc., Chicago, IL, USA). One‐way analysis of variance followed by Scheffe's post‐hoc test was performed to evaluate statistical significance (*P* < 0.05).

## 3 RESULTS

### 3.1 Significantly higher PSG10P and IL1RAP levels but lower miR‐19a‐3P levels are present in PE placentas compared to levels in normal placentas

**Figure 1:**
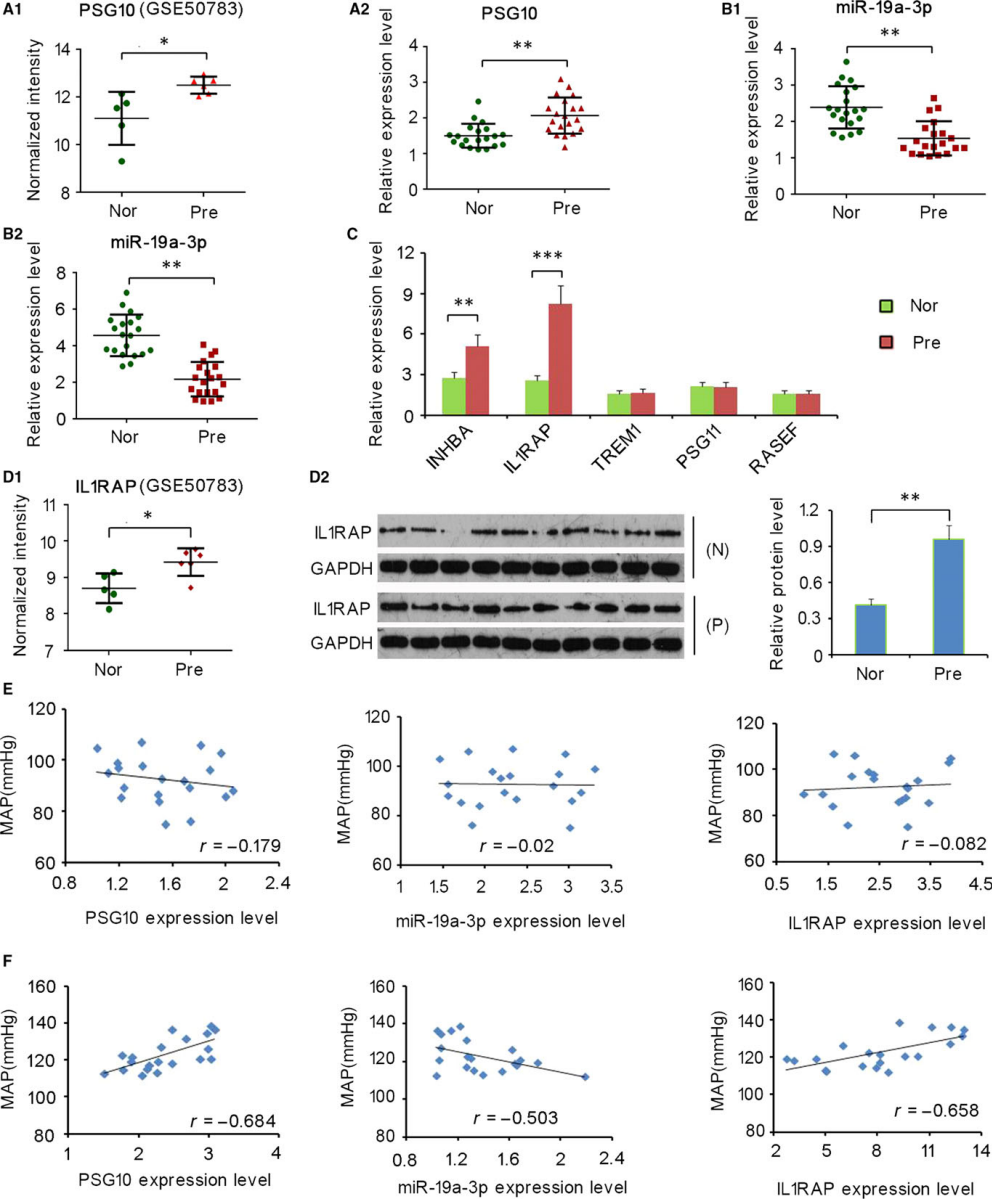
Expression profiles of PSG10P, miR‐19a‐3p, and IL1RAP in placentas of patients with pre‐eclampsia (PE) and controls and their association with mean arterial pressure (MAP). Expression profiles of PSG10P (A1) and IL1RAP (D1) in GSE50783 datasets were analysed. RT‐PCR was performed to detect the expression of PSG10P (A2), miR‐19a‐3p (B1), and a series of mRNAs (C) in placentas of patients with PE and controls. miR‐19a‐3p expression was also detected in the serum samples using RT‐PCR (B2). Western blotting was performed to detect the expression of IL1RAP in placentas of patients with PE and controls (D2). Association of the relationship between MAP and relative RNA expression of PSG10P, miR‐19a‐3p, and IL1RAP was analysed by Pearson correlation analysis in patient with PE (F) and normal controls (E). Nor and N:Normal group; Pre and P: Pre‐eclampsia group; MAP: Mean arterial pressure. *P < 0.05, **P < 0.01, ***P < 0.001 vs Control

GSE50783 datasets revealed a significantly higher expression level of PSG10 in PE placentas than in normal placentas (*P* < 0.05, Figure 1A1),^19^ which was also identified in the present study using more tissue samples (*P* < 0.01, Figure 1A2), but PSG10 was not detectable in the serum from either patients with PE or healthy controls (data not shown). miR‐19a‐3p, which was previously reported to be down‐regulated in PE placentas,^14^ was predicted to bind PSG10. The present study not only identified the down‐regulation of miR‐19a‐3p in PE placentas (*P* < 0.01, Figure 1B1), but also found a dramatic reduction in the serum of patients with PE (*P* < 0.01, Figure 1B2). From GSE50783 datasets, we found that five up‐regulated mRNAs (*INHBA*,* IL1RAP*,* TREM1*,* PSG11*, and *RASEF*) in PE placentas were targeted by miR‐19a‐3p. However, our PCR measurement only showed the up‐regulation of *INHBA* (*P* < 0.01) and *IL1RAP* (*P* < 0.001, Figure 1C), with the greatest increase in *IL1RAP*. Figure 1D1 shows the expression profile of *IL1RAP* in GSE50783 datasets. IL1RAP protein level was further assessed using western blotting. In agreement with the mRNA expression analysis, IL1RAP protein levels were increased in the PE placentas compared to that in normal placentas (*P* < 0.01, Figure 1D2).

### 3.2 Mean arterial pressure (MAP) is significantly correlated with levels of PSG10P, miR‐19a‐3P, and IL1RAP in patients with PE

As reported in a previous study, patients with PE show elevated systolic pressure and diastolic pressure.^1^, ^2^ Correlations between MAP of patients with PE and levels of PSG10P, miR‐19a‐3P, and IL1RAP were evaluated in this study. MAP showed a significant correlation with all three genes/proteins (all *P* < 0.05) in patients with PE (Figure 1F), but the correlation in healthy controls was poor (Figure 1E).

### 3.3 Trophoblast cells contain higher PSG10P and IL1RAP levels but lower miR‐19a‐3P levels under hypoxia than their expression under normoxia

**Figure 2:**
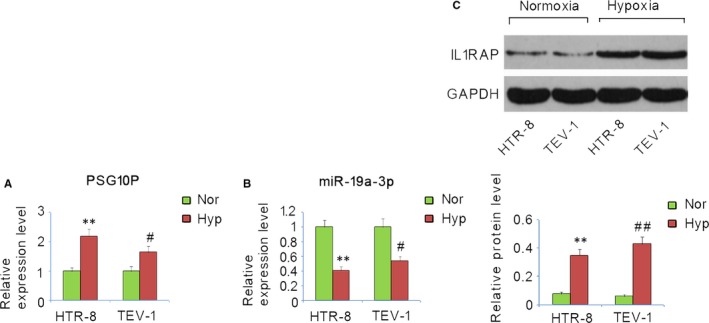
Expression profiles of PSG10P, miR‐19a‐3p, and IL1RAP in trophoblast cells under hypoxia and normoxia. HTR‐8/SVneo and TEV‐1 cells were cultured under hypoxia or normoxia. Expression profiles of PSG10P (A), miR‐19a‐3p (B), and IL1RAP (C) in trophoblast cells were assessed by RT‐PCR and western blot assays. Hyp: hypoxia; Nor: normoxia. *P < 0.05, **P < 0.01 vs HTR‐8/SVneo cells under normoxia; ^#^ *P* < 0.05, ^##^ *P* < 0.01 vs TEV‐1 cells under normoxia

HTR‐8/SVneo and TEV‐1 cells were cultured under low‐oxygen conditions to mimic the growth of trophoblasts under the hypoxic status in placentas of patients with PE. Under hypoxic conditions, significantly higher levels of lncRNA‐PSG10P and IL1RAP, as well as significantly lower levels of miR‐19a‐3P were detected (Figure 2A, B and C), which is consistent with results observed in PE placentas compared to normal placentas.

### 3.4 Alterations of PSG10P, miR‐19a‐3P, and IL1RAP expression in trophoblast cells after transfection

**Figure 3:**
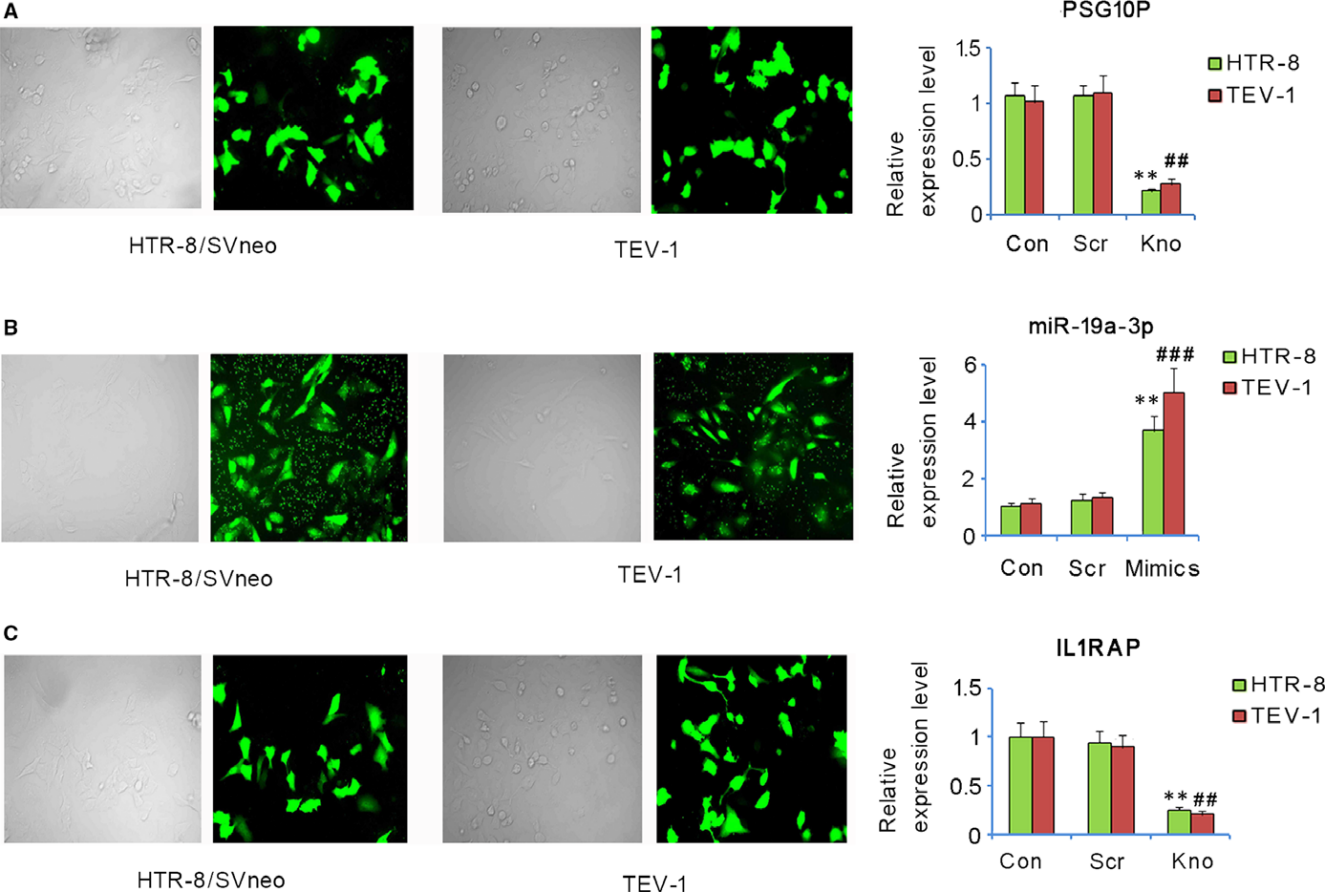
Changes of PSG10P, miR‐19a‐3P, and IL1RAP expression in trophoblast cells after transfection. HTR‐8/SVneo and TEV‐1 cells were transfected with shRNA‐PSG10P (A), hsa‐miR‐19a‐3p mimics (B), shRNA‐IL1RAP (C), and their negative controls. The transfection rate was determined by light microscopy and fluorescent images. PSG10P, miR‐19a‐3P, and IL1RAP expression were assessed by PT‐PCR. Con: control; Scr: scramble; Kno: knockdown of PSG10P or IL1RAP. *P < 0.05, **P < 0.01 vs HTR‐8/SVneo cells in control; ^#^ *P* < 0.05, ^##^ *P* < 0.01, ^###^ *P* < 0.001 vs TEV‐1 vs TEV‐1 cells in control

HTR‐8/SVneo and TEV‐1 cells were transfected with shRNA‐PSG10P, miR‐19a‐3p mimics, and shRNA‐IL1RAP to alter their expression. Transfection rates were more than 80% as indicated by the light microscopy and fluorescent images (Figure 3A, B, and C). RT‐qPCR analysis showed that transfection with shRNA‐PSG10P reduced PSG10P expression in both HTR‐8/SVneo and TEV‐1 cells compared to that in the negative control (both *P* < 0.01, Figure 3A). However, no change in the expression level was observed upon scramble treatment, confirming the successful knockout of PSG10P. Transfection with miR‐19a‐3p mimics increased miR‐19a‐3p expression in HTR‐8/SVneo (*P* < 0.01) and TEV‐1 cells (*P* < 0.001) compared to the negative control. shRNA‐IL1RAP transfection decreased IL1RAP expression in HTR‐8/SVneo and TEV‐1 cells (both *P* < 0.01).

### 3.5 PSG10P and miR‐19a‐3p expression are negatively correlated in trophoblast cells

**Figure 4:**
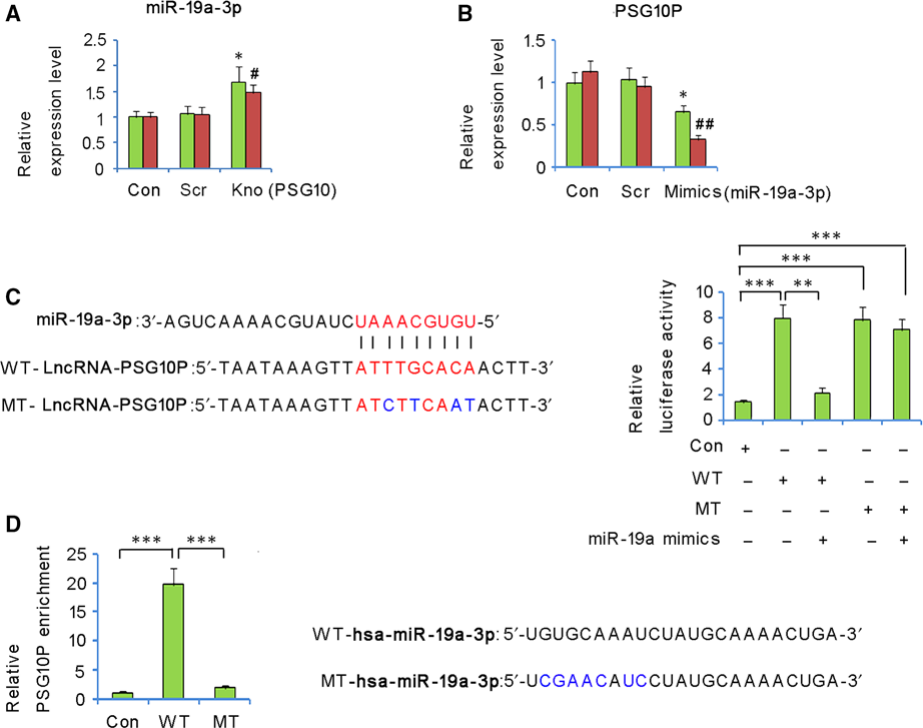
Negative correlation between PSG10P and miR‐19a‐3p expression in trophoblast cells. miR‐19a‐3p expression in trophoblast cells was analysed by RT‐PCR after PSG10P knockdown (A); PSG10P expression in the trophoblast cells was analysed by RT‐PCR after transfection with miR‐19a‐3p mimics (B). *P < 0.05, **P < 0.01 vs HTR?8/SVneo cells in control; ^#^ *P* < 0.05, ^##^ *P* < 0.01 vs TEV?1 cells in control. In a luciferase reporter assay, transfection with miR‐19a‐3p mimics significantly reduced the luciferase activity of the wild‐type of PSG10P constructs, but not that of the mutant PSG10P constructs (C). RNA pull‐down analysis indicated that PSG10P interacted with WT‐hsa‐miR‐19a‐3p, but not with MT‐hsa‐miR‐19a‐3p (D). *P < 0.05, **P < 0.01, and ***P < 0.001

PSG10P knockout was associated with a remarkable increase in miR‐19a‐3p expression in both HTR‐8/SVneo and TEV‐1 cells (both *P* < 0.05, Figure 4A). Transfection with miR‐19a‐3p mimics conversely decreased PSG10P expression in HTR‐8/SVneo (*P* < 0.05, Figure 4B) and TEV‐1 cells (*P* < 0.01).

The bioinformatic tools (Targetscan, miRanda, and PicTar) predicted that PSG10P is a direct target of miR‐19a‐3p and showed a putative complementary region. In luciferase reporter assays, both wild‐type and mutant‐type of PSG10P constructs showed increased luciferase activity compared to that of the negative control (*P* < 0.001, Figure 4C). However, transfection with miR‐19a‐3p mimics significantly reduced the luciferase activity of the wild‐type of PSG10P constructs (*P* < 0.01), but not that of the mutant type of PSG10P constructs, which confirmed the prediction of the target region. In addition, the interaction between miR‐19a‐3p and PSG10P was also identified by RNA pull‐down assay: significantly elevated relative PSG10P enrichment was only observed in wild‐type miR‐19a‐3p, but not in the mutant (Figure 4D).

### 3.6 IL1RAP is a direct target of miR‐19a‐3p

**Figure 5:**
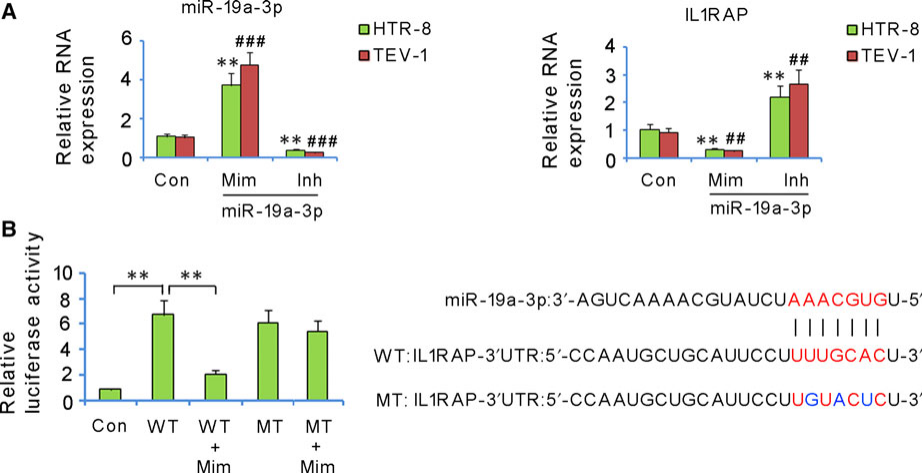
IL1RAP is a direct target of miR‐19a‐3p. miR‐19a‐3p and IL1RAP expression in trophoblast cells was assessed after transfection with miR‐19a‐3p mimics (Mim) and inhibitors (Inh) (A). *P < 0.05, **P < 0.01 vs HTR‐8/SVneo cells in control; ^#^ *P* < 0.05, ^##^ *P* < 0.01, ^###^ *P* < 0.001 vs TEV‐1 cells in the control. In the luciferase reporter assay, transfection with miR‐19a‐3p mimics significantly reduced the luciferase activity of the wild‐type of IL1RAP constructs, but not that of the mutant IL1RAP constructs (B). **P < 0.01

The mimics of miR‐19a‐3p significantly increased its relative RNA expression, while the inhibitors significantly inhibited its expression (Figure 5A). However, opposite expression behaviours were detected for IL1RAP; significantly lower relative RNA expression of IL1RAP was observed upon miR‐19a‐3p mimics treatment, but higher expression was observed in inhibitor treatment (Figure 5B). The putative binding site of miR‐19a‐3p in the 3′‐UTR region of IL1RAP is shown in Figure 5C. The transfection with miR‐19a‐3p mimics significantly reduced the luciferase activity of the wild‐type of IL1RAP 3′UTR constructs (*P* < 0.01, Figure 5D), but not that of the mutant‐type.

### 3.7 PSG10P, miR‐19a‐3P, and IL1RAP changed the growth and invasion properties of trophoblast cells under hypoxia

**Figure 6:**
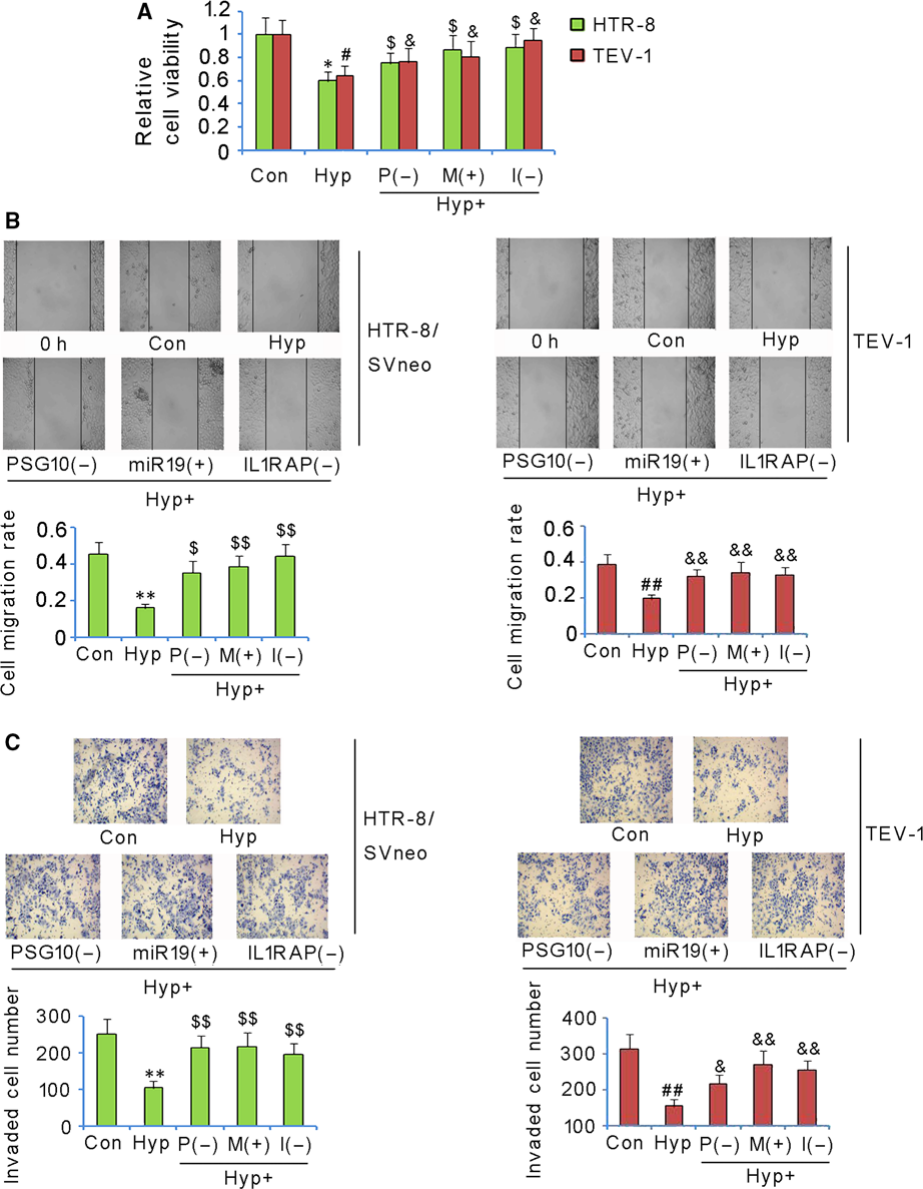
PSG10P, miR‐19a‐3P, and IL1RAP changed the growth and invasion properties of trophoblast cells under hypoxia. HTR‐8/SVneo and TEV‐1 cells were transfected with shRNA‐PSG10P, hsa‐miR‐19a‐3p mimics, or shRNA‐IL1RAP under hypoxia. After 48 h, cell viability (A), migration rate (B), and invasion rate (C) of both cell lines were evaluated. PSG10 (−) and P (−): PSG10P knockdown; miR19 (+) and M (+): miR‐19a‐3p overexpression; I (−) and IL1RAP (−): IL1RAP knockdown. *P < 0.05, **P < 0.01 vs HTR‐8/SVneo cells in control; ^$^ *P* < 0.05, ^$$^ *P* < 0.01 vs HTR‐8/SVneo cells under hypoxia. ^#^ *P* < 0.05, ^##^ *P* < 0.01 vs TEV‐1 cells in control; ^&^ *P* < 0.05, ^&&^ *P* < 0.01 vs TEV‐1 cells under hypoxia

HTR‐8/SVneo and TEV‐1 cells showed notably decreased cell viability after culture under hypoxia for 48 hours in comparison to that under normoxia (vs control group, *P* < 0.05, Figure 6A). However, interruption of PSG10P and IL1RAP as well as the overexpression of miR‐19a‐3p rescued the cell viability (vs Hyp group, *P* < 0.05). Cultivation under hypoxia for 48 hours also attenuated the migration and invasion capacity of HTR‐8/SVneo and TEV‐1 cells (vs control group, *P* < 0.05, Figure 6B and C). Interruption of both PSG10P and IL1RAP had a similar effect as the overexpression of miR‐19a‐3p: they all significantly promoted cell migration and cell invasion under hypoxia (vs hypoxic group, *P* < 0.01).

### 3.8 Increased miR‐19a‐3p expression rescued PE symptoms in vivo

**Figure 7:**
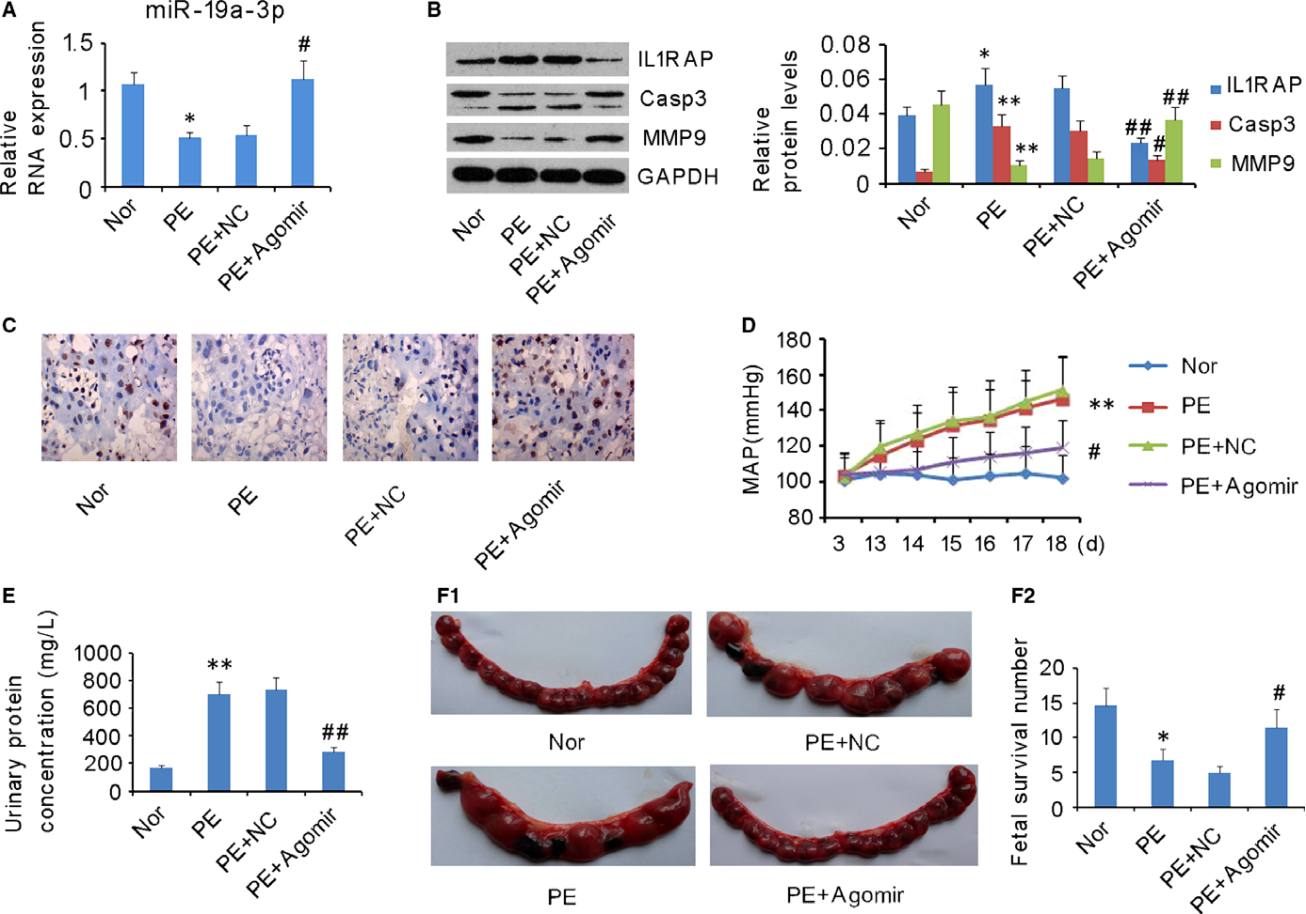
Increased miR‐19a‐3p expression rescued pre‐eclampsia (PE) symptoms in a mouse model. A mouse model of PE was established using sFlt‐1. Among 36 PE mice, 12 mice were randomly selected for mmu‐miR‐agomir treatment as a negative control (PE+NC), 12 for mmu‐miR‐19a‐3p agomir treatment (PE+Agomir), and 12 as a PE control (PE). In addition, another 12 normal pregnant mice were orally treated with saline solution as a control (Nor). (A) miR‐19a‐3p expression in placentas was assessed via RT‐PCR. (B) Protein levels of IL1RAP, caspase‐3, and MMP‐9 in placentas were assessed via western blot assay. (C) Ki67 expression in placentas was assessed via immunohistochemical staining. (D) The blood pressure, (E) urine protein concentrations, and (F) fetal survival number of the mice were recorded. *P < 0.05, **P < 0.01 vs Normal group; ^#^ *P* < 0.05, ^##^ *P* < 0.01 vs PE group

The mouse model of PE was established using sFlt‐1. From day 13, MAP significantly increased and reached as high as 140 at day 18 in the PE pregnant mice (*P* < 0.01, Figure 7D), urinary protein concentrations in the PE pregnant mice were dramatically increased (*P* < 0.05, Figure 7E), and PE pregnant mice showed reduced fetal survival number (*P* < 0.05, Figure 7F). miR‐19a‐3p expression was decreased in the placenta of PE pregnant mice (*P* < 0.05, Figure 7A). Injection of mmu‐miR‐19a‐3p reversed the miR‐19a‐3p expression (vs PE group, *P* < 0.05, Figure 7A), but injection of mmu‐miR‐NC had no such effect. PE pregnant mice showed increased IL1RAP and activated caspase‐3 levels (*P* < 0.05 and *P* < 0.01, respectively, Figure 7B), but decreased MMP‐9 (*P* < 0.01, Figure 7B) and Ki‐67 (Figure 7C) levels in the placenta. The injection of mmu‐miR‐19a‐3p, but not mmu‐miR‐agomir, decreased protein levels of IL1RAP, and activated caspase‐3 (vs PE group, *P* < 0.01 and *P* < 0.05, respectively), but increased the MMP‐9 level (vs PE group, *P* < 0.01) and Ki‐67 in the PE pregnant mice. Importantly, the injection of mmu‐miR‐19a‐3p recovered urinary protein concentration (vs PE group, *P* < 0.01, Figure 7E) and MAP (vs PE group, *P* < 0.05, Figure 7D), as well as improved fetal survival number (vs PE group, *P* < 0.05, Figure 7E) in the PE pregnant mice.

## 4 DISCUSSION

Many lncRNAs and miRNAs are aberrantly expressed in the placenta of patients with PE according to several comparative analyses between PE placentas and normal placentas, but reasons behind their abnormal expression, as well as the relationship between lncRNAs and miRNAs and their involvement in the PE pathogenesis are largely unknown. The present study found that the MAP of patients with PE was negatively correlated to the PSG10P expression, but positively correlated to miR‐19a‐3p expression in the placenta of patients with PE. Unfortunately, PSG10P and miR‐19a‐3p were up‐regulated and down‐regulated, respectively, in the placenta of patients with PE, as demonstrated in our study and others.^19^ The abnormal expression profiles are likely responsible for the elevated MAP in patients with PE. Our in vitro study found that hypoxia is an important driver of abnormal expression profiles. Moreover, miR‐19a‐3p was down‐regulated in the placentas of a pregnant mice model of PE which was established by injecting with sFlt‐1. It is known that sFlt‐1 can cause vascular endothelial dysfunction and further induce many abnormal symptoms commonly observed in PE, such as oxidative stress, imbalanced inflammatory response, hypotension, and proteinuria.^26^, ^27^

The relationship between PSG10P and miR‐19a‐3p was identified in this study by a series of analyses. The bioinformatic tools (Targetscan, miRanda, and PicTar) showed a putative complementary region between PSG10P and miR‐19a‐3p. Both luciferase reporter and RNA pull‐down assays demonstrated a mutual binding of PSG10P and miR‐19a‐3p in the cells. A negative correlation between PSG10P and miR‐19a‐3p expression was observed after PSG10P knockdown or miR‐19a‐3p overexpression. lncRNAs commonly function as an endogenous competitive RNA that sponges miRNAs and thus inhibits miRNAs from targeting mRNA. Accumulating evidence has indicated that lncRNA/miRNA networks participate in PE pathogenesis. The interaction between lncRNAs and miRNAs finally induces the altered mRNA levels. This is an important mechanism by which lncRNA/miRNA networks affect the occurrence and development of PE.

MiR‐19a was reported to significantly regulate cell proliferation and migration. miR‐19a‐3p inhibits invasion, migration, and bone metastasis in prostate cancer,^28^ while in lung cancer, miR‐19a promotes cell proliferation and migration.^29^ miR‐19a‐3p also regulates the viability, migration, and invasion of trophoblast cells, because the overexpression of miR‐19a‐3p attenuated the inhibition of the cell viability, migration, and invasion under hypoxemia. Trophoblast cells commonly show decreased viability, migration, and invasion in patients with PE, which results in inadequate vascular remodeling and consequent reduction of RUPP.^6^ Reduced RUPP has been confirmed as a leading cause for oxidative stress and over‐activation of the inflammatory response.^7^ Therefore, the improved cell viability and invasion capacity of trophoblast cells became very important to attenuate or prevent the adverse reactions in PE. Decreased expression of miR‐19a‐3p was observed in the placentas of patients with PE, PE pregnant mice, as well as in trophoblast cells under hypoxia. The overexpression of miR‐19a‐3p improved the cell viability, migration, and invasion under hypoxaemia in our in vitro study. Moreover, in the placentas of PE pregnant mice, forcing miR‐19a‐3p expression increased the cell proliferation (indicated by Ki67 expression), decreased apoptosis (indicated by caspase‐3 expression), and enhanced the invasion (indicated by MMP‐9 expression), which is associated with reduced MAP and urinary protein concentration, as well as increased fetal survival.

IL‐1, a pleiotropic cytokine, can activate the transcription of numerous genes and synthesis of a range of proteins involved in immunological responses.^30^ The critical role of IL1RAP in IL‐1 signal transduction suggests the involvement of IL1RAP in various inflammatory and immune responses affected by IL‐1. A previous report showed that a higher expression level of IL1RAP was associated with increased neutrophil counts in the airways of asthmatic patients, suggesting enhanced neutrophilic inflammation.^31^ The expression level of IL1RAP is likely associated with uterine sensitivity to IL‐1 during late gestation and delivery.^32^ IL1RAP is abnormally up‐regulated in the placentas of pregnant women with sickle cell anaemia [33]. Sickle cell disease is often accompanied with acute and chronic inflammation [33]. Our results also showed significantly higher IL1RAP levels in PE placentas than in normal placentas. We found that both PSG10P and IL1RAP were targets of miR‐19a‐3p. The presence of PSG10P could potentially sequester miR‐19a‐3p, resulting in accumulated free IL1RAP in patients with PE or associated transfected cell lines. Therefore, the higher PSG10P level could be a potential contributor to the lower level of miR‐19a‐3p. As a result, IL1RAP was up‐regulated, promoting the systemic maternal inflammatory response, which is characteristic in patients with PE. Additionally, IL1RAP also involved in regulating cell growth and invasion properties, as demonstrated by our in vitro study. Caspase‐3 is a caspase protein with a significant role in cell apoptosis, while MMP9 is involved in degrading the extracellular matrix, resulting in altered migration and invasion ability. A higher level of IL1RAP was always associated with more active caspase‐3 and lower MMP9, indicating that cell death was promoted and cell migration was inhibited. As mentioned, the capabilities of trophoblasts, including proliferation, migratory, and apoptosis properties, are essential for adequate placental formation and facilitating normal pregnancy. Our results strongly demonstrated that lncRNA‐PSG10P/miR‐19a‐3p/IL1RAP cooperate with each other as a regulatory network to manipulate CTBs during pregnancy.

There are some shortcomings of the present study. The potential effects of up‐regulated IL1RAP on the inflammatory response in patients with PE have not been discussed in the present study, although the pro‐inflammatory role of IL1RAP has been shown in other pathological models. In addition, the underlying mechanisms by which IL1RAP modulates the viability, migration, and invasion of trophoblasts under hypoxemia remain unclear. Therefore, all these questions should be answered in a further study.

In summary, miR‐19a‐3p, lncRNA‐PSG10P, and IL1RAP are involved in PE pathogenesis. With a common targeting region in their sequences, a regulatory network in the lncRNA‐PSG10P/miR‐19a‐3p/IL1RAP pathway may contribute to PE pathogenesis during pregnancy.

## DISCLOSURE OF INTERESTS

The authors declare that they have no conflict of interest.

## CONTRIBUTION TO AUTHORSHIP

Nan Wang and Ruizhen Li performed the experiment and wrote the manuscript. Min Xue took the responsibility of the line‐editing and proofreading.

## DETAILS OF ETHICS APPROVAL

All protocols used in this study were approved by the Research Medical Ethics Committee of the third Xiangya Hospital of Central South University. All women were informed of the research nature of our study with informed consent signed. The animal experiment was approved by the Ethical Committee for Animal Research of Central South University and adhered to National Institutes of Health Guidelines for the care and use of animals.
